# Use of autologous bone marrow stem cell implantation for osteonecrosis of the knee in sickle cell disease: a preliminary report

**DOI:** 10.1186/s12891-018-2067-x

**Published:** 2018-05-22

**Authors:** Gildasio Daltro, Bruno Adelmo Franco, Thiago Batista Faleiro, Davi Araujo Veiga Rosário, Paula Braga Daltro, Roberto Meyer, Vitor Fortuna

**Affiliations:** 1 Prof. Edgar Santos Hospital Complex, Federal University of Bahia, R. Doutor Augusto Viana, s/n - Canela, Salvador, BA 40110-060 Brazil; 2 Health Science Institute, Federal University of Bahia, Av. Reitor Miguel Calmon, s/n, Vale do Canela, Salvador, BA 40110-100 Brazil

**Keywords:** Osteonecrosis, Avascular necrosis, Knee, Sickle cell disease, Joint preserving surgery, Cell implantation

## Abstract

**Background:**

The purpose of our study was to evaluate safety, feasibility and clinical results of bone marrow mononuclear cell (BMC) implantation for early-stage osteonecrosis of the knee (OK) secondary to sickle cell disease.

**Methods:**

Thirty-three SCD patients (45 knees) with OK treated with BMC implantation in the osteonecrotic lesion were clinically and functionally evaluated through the American Knee Society Clinical Score (KSS), Knee Functional Score (KFS) and Numeric Rating Scale (NRS) pain score. MRI and radiographic examinations of the knee were assessed during a period of five years after intervention.

**Results:**

No complications or serious adverse event were associated with BMC implantation. From preoperative assessment to the latest follow-up, there was a significant (*p* < 0.001) improvement of clinical KSS (64.3 ± 9.7, range: 45–80 and 2.2 ± 4.1, range: 84–100, respectively), KFS (44.5 ± 8.0, range: 30–55 and 91.6 ± 5.8, range: 80–100, respectively) and reduction of NRS pain score (6.7 ± 1.2, range: 4–9 and 3.4 ± 1.0, range: 2–5, respectively). In total, 87% of patients (29/33) consistently experienced improvements in joint function and activity level as compared to preoperative score. No patient had additional surgery following BMC implantation. Radiographic assessment showed joint preservation and no progression to subchondral collapse at most recent follow-up.

**Conclusions:**

The technique of BMC implantation is a promising, relatively simple and safe procedure for OK in SCD patients. Larger and long-term controlled trials are needed to support its clinical effectiveness.

**Trial registration:**

ClinicalTrials.gov NCT02448121. Retrospectively registered 19 May 2015.

## Background

Osteonecrosis of the knee (OK) secondary to sickle cell disease (SCD) is a debilitating disorder that may progress to subchondral collapse and end-stage arthritis of the knee at an early age. Intravascular erythrocyte sickling and vaso-occlusion of microcirculation lead to ischemic death and necrosis of the constituents of the bone and marrow. In its early stage, the overlaying articular cartilage is intact, however, the changes in the subchondral bone may rapidly progress to structural collapse, chronic pain and joint destruction [1–3]. Advanced OK-SCD eventually requires knee arthroplasty, which is associated with increased risk of adverse outcome and higher revision rates in sickle cell disease patients [4]. For these reason, the early treatment is very important.

OK-SCD is usually part of a multifocal disease, and the knee is a particularly vulnerable joint in sickle cell disease. There have been a limited number of OK-SCD case reports and a high occurrence of osteonecrosis of the knee in asymptomatic patients [2, 5], therefore the exact occurrence of OK in sickle cell disease patients remains unclear. Flouzat-Lachaniete et al. [6] reported in a large study that 34% of SCD patients diagnosed with multifocal osteonecrosis had involvement of the knee. They also observed an annual incidence rate of 3.6 cases per 100 patients, which makes joint-preserving techniques important to initially attempt.

Treatment options for osteonecrosis of the knee in SCD patients are limited. For symptomatic patients at an early stage, many conservative procedures are used to enable knee preservation instead of replacing it [7]. However, the efficacy of these joint-preserving techniques, including core decompression or debridement of the osteonecrotic lesion, has been controversial in SCD and many patients still progress to advanced osteonecrosis [8–10]. Therefore, even if positive results are obtained, the treatment of the OK-SCD continues to be a daunting problem for orthopedic surgeons.

Cell therapy with autologous implantation of concentrated bone-marrow mononuclear cells (BMC) has been used successfully for treatment of hip osteonecrosis [11, 12]. Although no reports exist on the implantation of BMC in the knee in SCD patients, several authors described that BMC implantation can significantly decrease the pain and other joint symptoms caused by osteonecrosis and delay, or avoid the progress of this disease toward higher osteonecrotic stages [13, 14]. The rationale for the use of cell therapy for osteonecrosis in sickle cell disease patients relies on the presence of bone marrow-derived mesenchymal stromal cells (MSCs) and endothelial progenitors cells (EPCs) in BMC, which may enhance the mechanism of blood vessel formation and improve tissue repair [12, 15]. Other nucleated cells (macrophages, T cells, B cells, dendritic cells, natural killer cells and neutrophils) are also present in BMC and once introduced into the osteonecrotic lesion may help improve pain and function [13–15]. After encouraging results were obtained in the hip, the same technique has been transferred to the knee joint in sickle cell disease patients.

In this prospective pilot study we aimed to evaluate safety, feasibility, clinical and radiological results after minimally invasive implantation of autologous bone marrow mononuclear cell (BMC) in OK lesions secondary to sickle cell disease.

## Methods

### Study participants and design

The National Committee of Ethics in Research (CONEP) and the Brazilian Ministry of Public Health approved this study protocol. All patients provided full informed consent before enrollment in the study.

Between December 2010 and December 2016, we reviewed the prospectively collected records of sickle cell disease patients who had been evaluated and treated for symptomatic hip osteonecrosis. Of the 243 SCD cases receiving treatment in the orthopedic department at our institution, fifty-one patients were diagnosed as having early-stage knee osteonecrosis secondary to sickle cell disease. We excluded 2 patients in early stages knee osteonecrosis without pain. We excluded 4 patients who had undergone hip replacement surgery. We also excluded a further seven patients because of advanced knee osteonecrosis and five patients referring hip pain diffusing to lower limb. We evaluated the remaining patients and 33 patients (45 knees) were included. All our patients presented with painful symptoms and the knee was the most symptomatic joint at the time of enrollment. At least one year after the treatment of foregoing hip osteonecrosis was required to enroll in this open-label, prospective study. Physically active patients underwent clinical examination and had radiographic and magnetic resonance imaging (MRI) for both knees regardless of whether the knee was symptomatic. We identified patients with early-stage (stage I or II) knee osteonecrosis based on the Modified Ficat and Arlet staging system adapted for the knee [16–18] with characteristic MRI findings [19]. This radiographic classification staging system defined stage-I disease as knees with no abnormality. Stage-II disease have cystic or osteosclerotic lesions, or both, with a normal contour of the distal part of the femur or the proximal part of the tibia, or both, and with no subchondral fracture or flattening of the articular surface. Stage-III knees have flattening of the weight-bearing contour of the condyle, or oval radiolucency in the subchondral area and Stage-IV knees have narrowing of the joint space with secondary osteoarthritic changes. The medial and lateral femoral condyles and the medial and lateral tibial plateaus were evaluated separately with use of this system (Table 1).

**Table 1:** Staging system for Osteonecrosis of the Knee (OK) in Sickle Cell Disease [17]

Stage	Radiological findings
1	Normal appearance, accompanied by pain
2	Cystic or osteosclerotic lesions, or both, with a normal contour of the distal part of the femur or the proximal part of the tibia, or both, and with no subchondral flattening of the articular surface.
3	Presence of crescent sign. Flattening of the condyle or collapse of the subchondral bone plate with formation of a calcified plate and a clear sclerotic halo. Slightly narrowed joint space.
4	Osteoarthritic changes, such as spur formation and osteosclerosis, with a shallow concave articular surface at the osteonecrotic region

Inclusion criteria consisted of the following: (1) diagnosis of knee osteonecrosis stages 1 or 2 (pre-collapse); (2) presence of symptomatic, insidious and progressive pain; (3) availability of 1- and 5-year follow-up assessments. Exclusion criteria were (1) an age younger than 18 years or older than 55 years; (2) radiologic diagnosis of advanced knee osteonecrosis (stage III or IV); (3) comorbidities with general medical conditions (e.g., diabetes or rheumatoid arthritis); (4) previous knee surgery or presence of total hip replacement on affected or contralateral limb; (5) presence of at least one of the following conditions: Intra-articular corticosteroid injection in the affected knee, diffuse or degenerative osteoarthritis, infection, knee trauma, fracture or deformity involving the knee, neoplastic disease, inflammatory arthritis, immunosuppressive therapy, alcoholism or nicotine abuse.

A total of twenty female (60.6%) and thirteen (39.4%) male were enrolled. The genotypes of the patients enrolled included 48.5% patients who are homozygous for the sickle-cell gene (hemoglobin SS, HBSS) and 51.5% of patients with heterozygous combination with haemoglobin (hemoglobin SC, HBSC). Their mean age at the time of surgery was 28.4 years (range 18–42), and the mean duration of symptoms before surgery was 6 months. The details and laboratory characteristics of the study subjects are shown in Table 2.

**Table 2:** Patient Demographic Characteristics at Baseline

Parameter	Value
N^o^ of patients studies	33
Age (years), mean §	28.4 (18–42)
Men, n (%)	39.4
Hematocrit (%) ± SD	21.9
Total hemoglobin (g/l)	6.9
Genotype (N^o^)	
HbSS	16
HbSC	17
N^o^ of patients with bilateral involvement	36.4% (12/33)
N^o^ of knees enrolled	45
Staging	
Stage 1	48.5% (21/45)
Stage 2	51.5% (24/45)
Localization	
Medial femoral condyle	42.3% (19/45)
Lateral femoral condyle	24.4% (11/45)
Medial tibial plateau	20.0% (9/45)
Lateral tibial plateau	13.3% (6/45)

Abbreviation: *N*^*o*^, number; *SD*, standard deviation

§ Range is shown in parenthesis

Clinical and laboratory results obtained when patients were at steady state, without evidence of acute infection or pain crisis

### Bone marrow mononuclear cell (BMC) concentrate

Bone marrow aspirate (~ 120 mL) was harvested, under general anesthesia, from the patients’ anterior iliac crest according previously described [12]. The depth and angle of the trocar was changed after each 5 ml of aspirated material to avoid hemodilution. The aspirated bone marrow was processed directly in the operating room, by removing most of the erythrocytes and plasma. A cell separator (SEPAX, Biosafe, Switzerland) consisting of a centrifuge and a disposable double chamber device, provided approximately 40 mL (fixed volume) of concentrate containing bone marrow mononuclear cells (BMC) after sixty minutes of multiple centrifugation, in accordance with the manufacturer’s recommendations. The BMC was transferred to a 20 mL syringe for injection into the osteonecrotic lesion. This procedure required around 2 h from bone marrow collection, extraction, processing and injection into the patient. A small fraction of the BMC was separated for flow cytometry, cell viability and microbiological assays.

### Operative technique

With the patient in the supine position, a 3-mm marrow-needle was inserted in the center of the osteonecrotic area under fluoroscopic guidance using a minimally invasive procedure. The BMC implantation was done through a percutaneous approach when the needle was advanced until it reached the lesion in the epiphyseal region. Each patient received the BMC implantation through a single injection puncture. The needle was directed to either the medial or lateral condylar lesion under fluoroscopic guidance and the BMC was injected at once into the osteonecrotic lesion. Coexisting injuries were treated concurrently as needed. Bleeding or leakage of the infused BMC fraction was controlled.

### BMC viability and flow cytometry analysis

The viability of infused cells, as determined by Trypan blue exclusion, was above 95%. The number of nucleated cells in each patient sample was counted with an automatic hemocytometer. Each sample was counted three times and the average calculated. The total nucleated cell count for injection was determined by multiplying the dilution factor and the final volume of the sample.

BMC were also analyzed by flow cytometry. The cell membrane antigens analyzed included those proposed by the ISHAGE protocol: anti-CD34-PE (BD Bioscience, NJ, USA), anti-CD45-FITC (Becton Dickinson, CA, USA) and their appropriate isotype-matched IgGs. For analysis, 5 × 10^5^ events were acquired and scored with a FACS Calibur analyzer (Becton Dickinson). Data were processed using the Macintosh CELLQuest software program (Becton Dickinson). CFU-F assays were done according to previously described [12].

### Clinical and radiographic examination

All patients were clinically evaluated before surgery, and at regular intervals after BMC implantation, by two independent examiners. Evaluation was scheduled at one month, one year and yearly thereafter.

The data were collected prospectively and included demographic, peri-operative data, and clinical outcome. Objective clinical and functional assessment was performed in a nonblinded manner by the treating surgeon using the American Knee Society scoring system, a dual rating system that comprises the Knee Society Score (KSS) and Knee Functional Score (KFS). The KSS includes pain, range of motion, flexion contractures, extension lag, alignment, and stability in the anteroposterior and mediolateral plane. The KFS includes the level of activity, walking/stair climbing and use of assistive devices [20, 21]. The results were both classified based on a 100-point scale as excellent (score, 85–100), good (score, 70–84), fair (score, 60–69), and poor (score, ≤ 59).

The patients were questioned concerning their pain, the type of onset (sudden or progressive), the main areas of pain together with the spread areas, the presence of limp, the history of a triggering event, and the duration of symptoms. Patients with moderate to severe hip pain, diffusing to lower limb were not included in the study. Patients were prospectively analyzed with standardized numeric rating scale (NRS) to evaluate the knee pain severity (where “0” represented *no pain*, and “10” represented *the maximum possible or unbearable pain*) and a NRS Satisfaction subscale to assess patient satisfaction with the surgery [22]. The distribution of knee pain was not significantly different when patients had foregoing treatment of hip osteonecrosis. Failure was defined as the need of a new surgical procedure to treat persisting pain or effusion in the previously operated knee.

Radiographs and MRIs of the affected knee were taken at presentation. At the most recent follow-up, radiographic assessment included evaluation for radiolucent lines and was performed using standing weight-bearing anteroposterior and lateral radiographs of the knee. Postoperative MRIs were not performed.

### Statistical analysis

Continuous data were described as mean ± SD and categorical variables as mode and range. The distribution of continuous data was determined using the Kolmogorov-Smirnov test. Comparisons between the pre-treatment and follow-up scores were made using Student’s t-test for independent continuous variables or the Mann–Whitney U-test and Wilcoxon sign-rank test when the assumption of normality was not realized. To assess the correlation between clinical findings (KSS / KFS) and categorical data, the differences between pre- and post-treatment values (change in KSS and change in KFS) were calculated. Then, continuous and categorical data were examined using the Pearson’s Chi-Square Test. The independent variables explored were gender, stage of the osteonecrosis, genotype of the sickle cell disease and presence of bilateral knee osteonecrosis. Statistical significance was defined as *P* < 0.05.

## Results

**Fig. 1:**
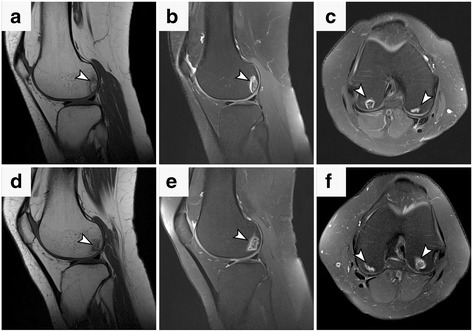
Osteonecrosis of the knees in a 33-year-old woman with SCD. (**a** - **f**) T1-weighted (**a**, **d** - sagittal) and proton density fat-suppressed (**b**, **e** - sagittal; **c, f** - axial) sequences demonstrates subchondral lesions (arrowheads) on the posterior portions of the right (**a**, **b**, **c**) and left (**d**, **e**, **f**) condyles. The osteonecrotic lesion is surrounded by border appearing hypo/hyperintense (double rim sign) (**b**, **c**, **e**, **f**) indicating granulation and sclerosis respectively (arrowheads). MRI scans before surgery shows subchondral lesions representing early osteonecrosis in Ficat II stage. Both knees were treated with BMC implantation

**Fig. 2:**
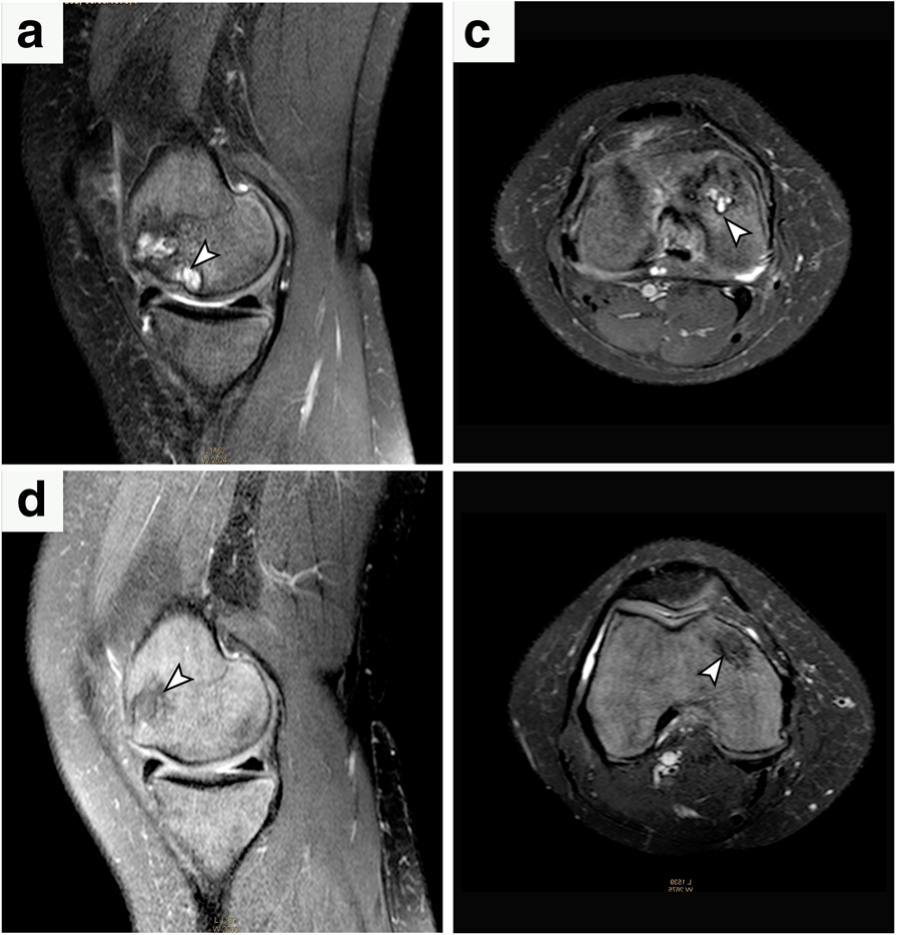
Bilateral osteonecrosis of the knees in a symptomatic 39-year-old woman with SCD. (**a** - **d**) Proton density fat-suppressed (**a**, **c** – sagittal) and T2-weighted (**b**, **d** - axial) sequences demonstrate subchondral abnormalities (arrowheads) located in the medial femoral condyle (weight bearing zones). Subcortical high-intensity inner line (**a** – **c**) representing granulation tissue. MRI scans before surgery shows osteonecrotic lesion in Ficat I and II on left and right knees, respectively. Both knees were treated with BMC implantation

At presentation, 21 patients (63.6%) had unilateral osteonecrosis (the right knee was affected in 9 cases and the left knee in 12 cases) and 12 patients (36.4%) had bilateral osteonecrosis. OK was stage I in 16 patients (21 knees) and stage II in 17 patients (24 knees), according to classification system (Table 1). Osteonecrotic lesions were predominantly located in the medial femoral condyle (19 knees, i.e. 42.3%) followed by the lateral femoral condyle (Table 2). Examination of preoperative MRI consistently revealed subchondral areas of early-stage osteonecrosis and diffuse bone marrow edema (Figs. 1 and 2).

Overall, the interventional procedure was well tolerated by SCD patients, with no complications or serious adverse event (deaths, hematoma, nerve injury and others). Only four episodes of small adverse events (site infection following cell injection, and transient local pain) were noted. No serious adverse events or failure led to treatment discontinuation or study termination. The mean observation follow up period was 27.3 months (range: 12–60).

**Fig. 3:**
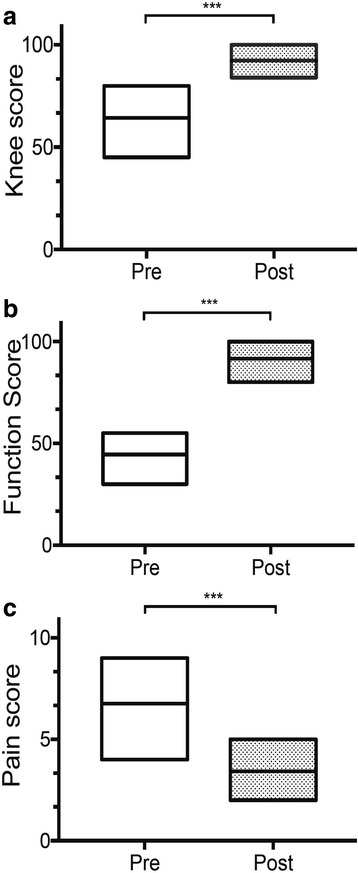
BMC treatment significantly improved both clinical and functional outcome of early stage OK-SCD patients (**a**, **b**) Preoperative and postoperative changes of Knee and functional score for patients grouped over the entire postoperative period. Floating bar represent max and min range of variation, with line at mean score. **c** Pain level was evaluated using the Visual Analog Scale. Most of the patients with pain preoperative showed a significant decrease in Pain Scoring *P < 0.05, **P < 0.01, ***P < 0.005, ****P < 0.001 versus preoperative score values (paired t-test)

**Fig. 4:**
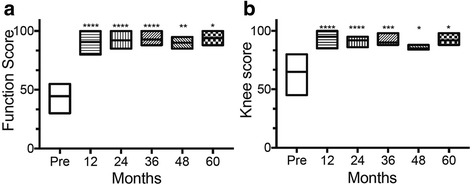
Functional and clinical improvement over time after BMC implantation. **a**, **b** Function score (**a**) and Knee score (**b**) evaluation preoperatively and at the time of follow-up showed increased and sustained clinical improvement over time. *P < 0.05, **P < 0.01, ***P < 0.005, ****P < 0.001 versus preoperative score values (paired t-test)

All SCD patients suffered from symptomatic knee joint and limitation of knee motion at the time of enrollment. Pre-operative KSS was poor in 8/33 patients (24.2%) and fair in 17/33 patients (51.6%) whereas KFS was poor in 31/33 patients (94%) and fair in 2/33 patients (6%) (Table 3). After the percutaneous BMC implantation improvements were observed in both KSS and KFS rating systems compared to the preoperative state. After surgery, 97% (32 out of 33 patients) had excellent outcome in KSS score whereas the KFS score was good in three patients (9%), and excellent in the remaining thirty patients (91%). The mean KSS (and standard deviation) was 64.3 ± 9.7 (range: 45–80) before surgery and 92.2 ± 4.1 (range: 84–100) at a mean of 27.3 ± 13.8 months (*p* < 0.001), while the KFS was 44.5 ± 8.0 (range: 30–55) before surgery and 91.6 ± 5.8 (range: 80–100) at a mean of 27.3 ± 13.8 months (p < 0.001) (Fig. 3a, b) (Table 3). KSS and KFS evaluation preoperatively and at the time of follow-up showed clinical improvement was maintained over time (Fig. 4a, b). However, no correlation was seen between change in clinical scores (changes in KSS or KFS) and gender (*p* = 0.02; 0.008), stage of the osteonecrosis (*p* = 0.26; 0.11), genotype of the sickle cell disease (*p* = 0.35; 0.12), and presence of bilateral knee osteonecrosis (0.015; 0.10), respectively.

**Table 3:** Overall Results Based on the American Knee Society Scoring System

Parameter	Preoperative	Postoperative	*P* value
Pain Score	6.7 ± 1.2	3.4 ± 1.0	0.001^*^
Knee Score (KSS)			
Average Score	64.3 ± 9.6	92.2 ± 4.2	0.001^†^
Excellent	0	97% (32/33)	
Good	24.2% (8/33)	3% (1/33)	
Fair	51.6% (17/33)	0	
Poor	24.2% (8/33)	0	
Function score (KSS)			
Average Score	44.5 ± 8.0	91.6 ± 5.7	0.001^†^
Excellent	0	91% (30/33)	
Good	0	9.0% (3/33)	
Fair	6% (2/33)	0	
Poor	94% (31/33)	0	

Values are presented as mean ± standard deviation

KSS: Knee Society score

*Wilcoxon signed-rank test. †Paired t-test

At beginning, all 33 patients had moderate or severe pain usually localized, occurring both at motion, weight-bearing and at rest, causing significant functional impairment in all cases. After BMC implantation, pain decreased was observed in 87.8% (29/33 patients) and remained unchanged only in 4 (12.2%) patients. The mean NRS pain score decreased from 6.7 ± 1.2 (mode: 7; range: 4–9) to 4.5 ± 0.8 (mode: 4; range: 2–5) after BMC implantation (*p* < 0.001) (Fig. 3c) (Table 3). Among 33 patients, 29 (87%) were highly satisfied with the surgical outcome, three (13%) were moderately satisfied. The mean NRS satisfaction score obtained was 9.2 at the most recent follow-up.

The total amount of BMC-nucleated cell ranged from 10 to 37 × 10^6^ cell/ml (range: 4.5–16.7 × 10^8^ nucleated cell/patient), including < 1.2% CD34+ CD45^dim^ positive cells, which are precursors of hematopoietic cells, and 2.1 +/− 1.4 × 10^4^ cells of CFU-F as an indicator of mesenchymal stromal cell activity. These results show that up to 19.9 × 10^6^ CD34+ CD45^dim^ progenitor cells were injected through and distributed into the ostenecrotic zones.

**Fig. 5:**
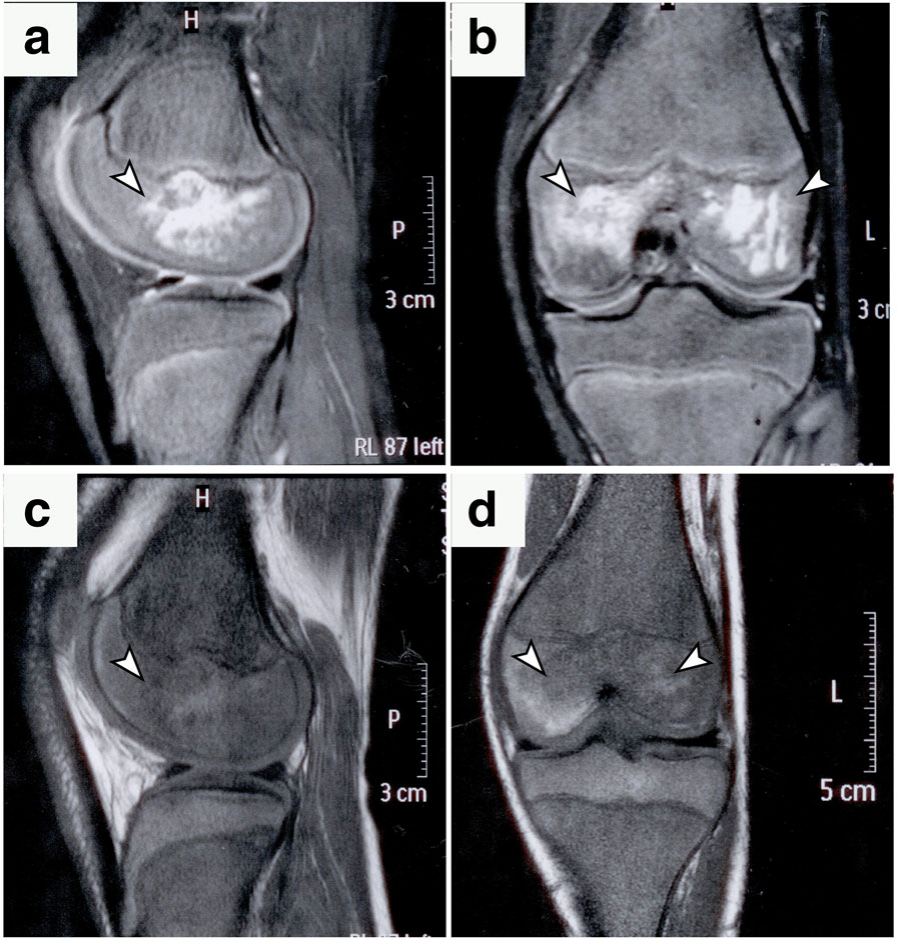
MR imaging after successfully treatment with BMC. **a** - **c** MR imaging of the 16-years-old boy before (**a**, **b**) and after surgery (**c**, **d**, 12 months postoperatively). PD-weighted (**a**, **b**) and T1-weighted (**c**, **d**) sequences demonstrate no subchondral bone erosion or fracture, normal contour of the epiphyseal head and preservation of the joint space. Proton density-weighted (**a**, **b**) sequences demonstrate mostly hematopoietic marrow without cystic or fibrotic lesion

**Fig. 6:**
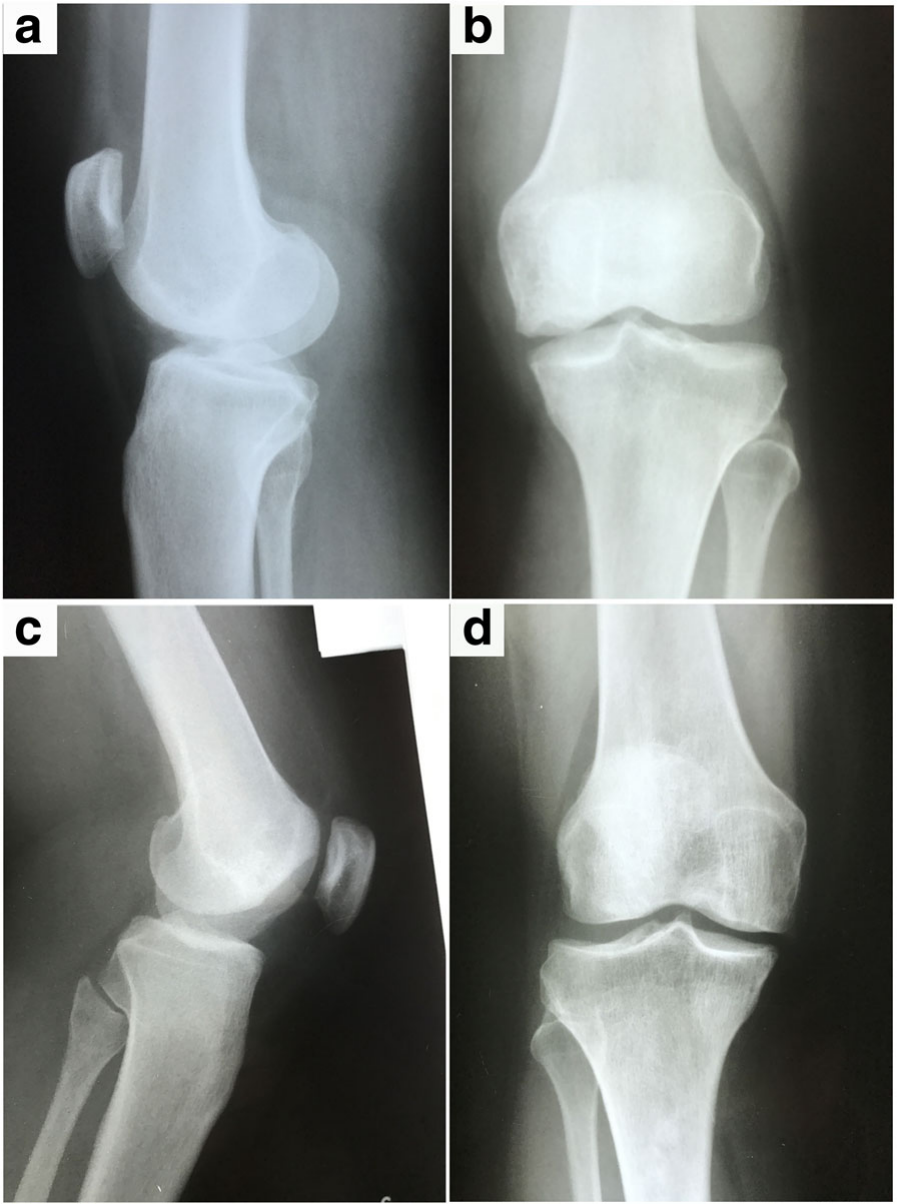
Radiographic imaging after successfully treatment with BMC. **a**, **b** Radiographs of the patient displayed on Fig. 1, 36 months postoperatively showing joint preservation and no subchondral bone fracture; (**c**, **d**) Radiographs of the patient displayed on Fig. 2, 60 months postoperatively. The joint space was preserved and the patient was asymptomatic ate final follow-up

In contrast to the improved clinical outcome, radiographic signs did not change appreciably after BMC implantation. We could not perceive any sign of subchondral fracture or collapse of the femoral condyles or the tibial plateau at the follow-up times after intervention. At the latest radiographic evaluation, any significant subchondral bone changes of radiographic signs, including presence of crescent sign, increased thickness, density, and irregularity were noted. Postoperative MRI scans demonstrated no subchondral bone erosion or fracture, normal contour of the epiphyseal head and preservation of the joint space. Most lesions that appeared stable on MRI were clinically also stable or improved. These results indicate that the joint space was preserved and the subchondral bone was stable after BMC implantation during follow-up period. Figs. 5 and 6 shows representative cases from our series.

## Discussion

The most important finding of the present study is that early stages of osteonecrosis of the knee in SCD patients achieved clinically meaningful and significant improvement after the implantation of BMC, compared to the preoperative situation. The results also demonstrated that BMC could be safely delivered in a concentrated manner to osteonecrotic lesions of the knee and no radiographic evidence of subchondral collapse at midterm follow-up (average: 27.3 months) in SCD patients.

Osteonecrosis of the knee in sickle cell disease is most commonly seen in patients who are younger than 45 years of age, with typical bilateral knee involvement. The medial and lateral femoral condyles are relatively frequent sites for its occurrence [6, 7]. As with osteonecrotic hip in sickle cell disease, variable mechanisms may be implicated in the knee, including interruption of the vascular blood supply to the subchondral bone. If not treated, early stages of OK-SCD have a high likelihood of progression to symptomatic disease and subchondral collapse, as demonstrated for hip, shoulder and ankle [2, 23, 24]. In these situations, conservative treatment is generally not successful, leading to secondary arthritis and total joint replacement in approximately 80% of cases after 2–5 years in SCD patients [2, 13].

Joint preservation therapy is the immediate goal of treatment considering the prevalence of OK-SCD among young adult patients in our series. Instead, joint replacement is a controversial option considering the risk and reduced durability of total knee arthroplasty in SCD patients. Recently, Perfetti et al. [4] have shown that sickle cell disease patients admitted for total knee arthroplasty have a 137% higher risk for perioperative complication than that of non-SCD patients. Moreover, joint replacement was associated with several complications in SCD patients: intra-operative bleeding, infections, and loosening or early loss of the prosthesis [25]. For a small and preliminary study as ours, in which the main focus was safety and feasibility, the results confirmed that our knee joint-preserving procedure was promising and had a limited rate of procedure-associated adverse effects. Therefore, we suggest that BMC implantation might be a valuable therapeutic joint preserving option for osteonecrosis of the knee in SCD patients.

Conventional treatments are unable to reverse or halt early stage osteonecrosis of the knee [26, 27] and its efficacy remains controversial for SCD patients [8, 9]. MRIs and morphological analysis have shown that no significant repair process in the osteonecrotic lesion could be achieved with core decompression alone [14], calling for additional alternative treatment options, usually with some form of bone grafting. In our series, we included SCD patients with OK in early pre-collapse stages, since its recognition is important for the long-term result of this procedure [11, 12].

Here, we treat osteonecrosis by implantation of autologous progenitor stem cells into the necrotic lesion. In sickle cell disease, implantation of BMC has been described for over twenty years with successful results for patients suffering with early stage osteonecrosis of the humeral and femoral head. [13]. This procedure has been used in the senior author’s practice for the past ten years for osteonecrosis of the femoral head, and the same technique has been transferred to the knee joint in sickle cell disease patients. The percutaneous minimally invasive technique in our study uses small-diameter marrow-needle as a modification of core decompression, proper patient immobilization and knee positioning, with minimal morbidity being associated with the procedure. Furthermore, surgery was performed carefully, with minimal trauma to avoid unexpected drilling through the articular cartilage surface or supracondylar femur fracture in OK-SCD patients.

Autologous concentrated bone marrow-based approaches for knee osteonecrosis have been limited to case reports or to osteonecrosis associated with other risk factors rather then sickle cell disease [27]. Recently, Goodman et al. [28] have shown that 12 young patients (fourteen knees) with OK associated to corticosteroid and submitted to local debridement before osteoprogenitor cell grafting had excellent pain relief and function, integration of the graft and no radiographic evidence of subchondral collapse at up to 5 years postoperatively. Marulanda et al. [26] reported a series of 61 knees (38 patients) with OK treated by a technique using multiple small percutaneous 3 mm drillings. A total of 56 (92%) had a successful clinical outcome, with 5 (8%) knees progressing to further surgery. Buda and colleagues [29] evaluated the midterm (average follow-up: 29 months) results of 30 patients with post-traumatic osteochondral lesions of the knee treated predominantly by arthroscopic transplantation of concentrated bone-marrow aspirate with a scaffold into the lesion site. They reported good to excellent results in all the patients based on the IKDC and KOOS scores, and growth of bone and cartilage, nearly complete defect filling and satisfactory integration of the graft at follow-up in 80% of cases [29]. Gobbi et al. [30] reported a long-term and sustained benefit of concentrated bone-marrow aspirate compared to microfracture for the treatment of osteochondral lesions of the knees. Zellner et al. [31] described promising clinical and radiological results, achieving successful bone regeneration of large and deep osteochondral defects of the knee joint after the treatment with concentrated bone-marrow aspirate combined with bone augmentation. In our study, arthroplasty was avoided in all the patients during the follow-up period. There was also significant improvement in the KSS, KFS and NRS pain score at the time of the latest follow up. Only 4 knees (out of 33) had persistent painful symptoms after treatment, which required pharmacological management. These outcomes are promising in that knee osteonecrosis can be safely and effectively treated with BMC implantation. Although these initial results seem quite encouraging, longer-term follow up is clearly needed.

Consistent with the previously published results from our group and others, significant improvement of pain was observed in knee joints following injection with BMC [12, 32]. Pain in the knee is often referred in patients with hip disorders [33]. However, patients were selected based on the knee pain, and the distribution of knee pain was not significantly different when patients had previous diagnosis of hip osteonecrosis. We suggest that SCD patients with knee osteonecrosis often present with pain because the main lesion is on the knee, especially in the medial femoral condyle (load transmitting area). Treatment of knee osteonecrosis with BMC showed only local improvement and no effect on the other joints, which is consistent with previous reports [30–32].

Cell therapy efficacy for osteonecrotic lesions depends on the quantity of implanted viable BM nucleated cells and stem cells progenitors [13]. Hematopoietic/endothelial progenitors (CD34CD45^low^), mesenchymal stromal cells (CFU-F) and nucleated cell into the osteonecrotic lesion may help by replenishing damaged joint structures and providing modulation of the immune response, thus alleviating the symptoms and progression of the disease [13, 14, 30]. However, the correlation between BMC implantation and clinical success is in open investigation. Prior studies investigating a relationship between the cellular dose contained within the bone marrow concentrate and efficacy of the treatment for knee osteoarthritis have shown that patients receiving a higher concentration of cells (< 10^8^ nucleated cell/patient) reported a better pain outcome in comparison with the lower dose group [32]. In our study, the cellular doses administered were adequately high above the BMC-nucleated cell indicated. Moreover, the frequency of hematopoietic/endothelial progenitors (CD34CD45^low^) and mesenchymal stromal cells (CFU-F) were consistent with those in the literature, and the number of CFU-F that was injected was in accordance with previous clinical studies (between 25,000 and 200,000 CFU-Fs) [13, 14]. Therefore, despite a low number of sample sizes tested, these results suggest that there is no deficiency in the quantity of grafted SCD patient’s marrow stromal cell populations.

We are conscious that our experimental design has several limitations. The presented data collection is not a controlled study. Sickle cell disease is a systemic life-threatening hemoglobinopathy associated with several comorbidities, and there is no current standard treatment for osteonecrosis in these patients. Also, the inclusion of a control group (placebo group) was not in accordance with ethical recommendations of the national authorities that have analyzed and provided the financial support for this study. Quite clearly, prospective randomized studies including a comparison with an active control group would be nevertheless desirable, in order to determine more comprehensively the efficacy of the treatment described in this study.

Our study has several others limitations: 1) The number of patients is small and the follow-up short to intermediate term; 2) We selected only patients who had associated osteonecrosis of the hip. Because there may be patients with knee osteonecrosis who do not have hip osteonecrosis, this study population might not represent all sickle cell patients with osteonecrosis of the knee; 3) We cannot be sure that implanted BMCs remained at the site of implantation during the follow up period; 4) Another limitation is consequent to the fact that only a few patients underwent MRI during the follow-up because of the costs associated with imaging asymptomatic patients with sickle cell disease. Monitoring bone formation in the necrotic area or examine how BMC implantation may have affected outcomes should be incorporated in future studies.

## Conclusion

Despite these limitations, implantation of BMC in young sickle cell disease patients with secondary osteonecrosis of the knee was relatively simple, promoted pain relief and functional improvement at midterm follow-up. This technique does not preclude further reconstructive operations, if necessary. This prospective pilot study suggest that the treatment strategy of BMC is a promising strategy and may be useful to treat early stage knee osteonecrosis in sickle cell disease patients without subchondral bone collapse and large degenerative changes of the cartilage.

## Abbreviations

BMC: Bone marrow mononuclear cells
CFU: Colony-forming units
EPC: Endothelial progenitors cell
HbS: Hemoglobin S
KSS: Knee Society Score
MRI: Magnetic Resonance Imaging
MSC: Bone marrow–derived mesenchymal stromal cell
OK: Osteonecrosis of the knee
SCD: Sickle cell disease
